# Nanoscale optical positioning of single quantum dots for bright and pure single-photon emission

**DOI:** 10.1038/ncomms8833

**Published:** 2015-07-27

**Authors:** Luca Sapienza, Marcelo Davanço, Antonio Badolato, Kartik Srinivasan

**Affiliations:** 1 Center for Nanoscale Science and Technology, National Institute of Standards and Technology, Gaithersburg, Maryland 20899, USA; 2 Maryland NanoCenter, University of Maryland, College Park, Maryland 20742, USA; 3 School of Physics and Astronomy, University of Southampton, Southampton SO17 1BJ, UK; 4 Department of Physics and Astronomy,University of Rochester, Rochester, New York 14627, USA

## Abstract

Self-assembled, epitaxially grown InAs/GaAs quantum dots (QDs) are promising semiconductor quantum emitters that can be integrated on a chip for a variety of photonic quantum information science applications. However, self-assembled growth results in an essentially random in-plane spatial distribution of QDs, presenting a challenge in creating devices that exploit the strong interaction of single QDs with highly confined optical modes. Here, we present a photoluminescence imaging approach for locating single QDs with respect to alignment features with an average position uncertainty <30 nm (<10 nm when using a solid-immersion lens), which represents an enabling technology for the creation of optimized single QD devices. To that end, we create QD single-photon sources, based on a circular Bragg grating geometry, that simultaneously exhibit high collection efficiency (48%±5% into a 0.4 numerical aperture lens, close to the theoretically predicted value of 50%), low multiphoton probability (*g*^(2)^(0) <1%), and a significant Purcell enhancement factor (≈3).

## Abstract (web summary)

Self-assembled quantum dots are good emitters, but lack emission control prior to device fabrication. Here a photoluminescence imaging technique to characterize position and emission properties of such quantum dots is demonstrated, enabling the realization of high-performance single-photon sources.

## Graphical abstract

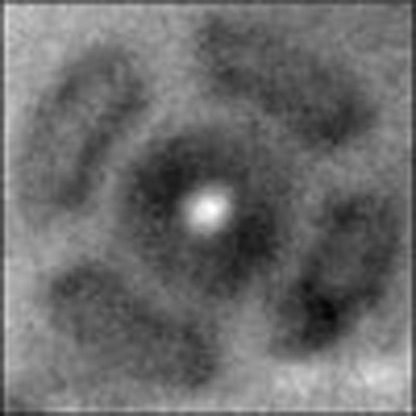

Single InAs/GaAs quantum dots (QDs) are one of the most promising solid-state quantum emitters for applications such as quantum light generation and single-photon level nonlinear optics^1^. Critical to many such applications is the incorporation of the QD within an engineered photonic environment so that the QD interacts with only specific optical modes. A variety of geometries such as photonic crystal devices and whispering gallery mode resonators have been employed to achieve such behaviour for bright single-photon sources and strongly-coupled QD-cavity systems^2^. The optical field in many such geometries varies significantly over distances of ≈100 nm, setting a scale for how accurately the QD position should be controlled within the device for optimal interaction. While site-controlled growth of QDs presents one attractive option^3^, the properties of such QDs (in terms of homogeneous linewidth, for example) have not yet matched those of QDs grown by strain-mediated self-assembly (Stranski–Krastanow growth)^4^. However, the in-plane location, polarization, and emission wavelength of such self-assembled QDs are not accurately controlled in a deterministic fashion, and thus techniques are required to determine these properties before device fabrication, in order to create optimally performing systems. Several techniques for location of self-assembled InAs/GaAs QDs before device fabrication have been reported, including atomic force microscopy (AFM)^5^, scanning confocal photoluminescence microscopy^6^ (including *in situ*, cryogenic photolithography^7^,^8^), photoluminescence imaging^9^, and scanning cathodoluminescence^10^. Of these approaches, photoluminescence imaging is particularly attractive given its potential to combine high throughput sub-50 nm positioning accuracy, spectral information, and compatibility with high-resolution electron-beam lithography that is typically used to pattern small features such as those used in photonic crystals. Localization of single molecules to 10 nm scale accuracy by imaging their fluorescence onto a sensitive camera has proven to be a powerful technique in the biological sciences^11^.

Here we present a two-colour photoluminescence imaging technique to determine the position of single QDs with respect to fiducial alignment marks, with an average position uncertainty <30 nm obtained for an image acquisition time of 120 s (the average position uncertainty is reduced to <10 nm when using a solid-immersion lens). This wide-field technique is combined with confocal measurements within the same experimental set-up to determine emission wavelength and polarization. We use this information to fabricate and demonstrate QD single-photon sources in a circular Bragg grating geometry that simultaneously exhibit high collection efficiency (48%±5% into a lens with numerical aperture of 0.4), low multiphoton probability at this collection efficiency (*g*^(2)^(0) <1%), and a significant Purcell enhancement factor (≈3). Our results constitute an important step forward for both the general creation of nanophotonic devices using positioned QDs, and the specific performance of QD single-photon sources.

## Results

### QD location via photoluminescence imaging

**Figure 1:**
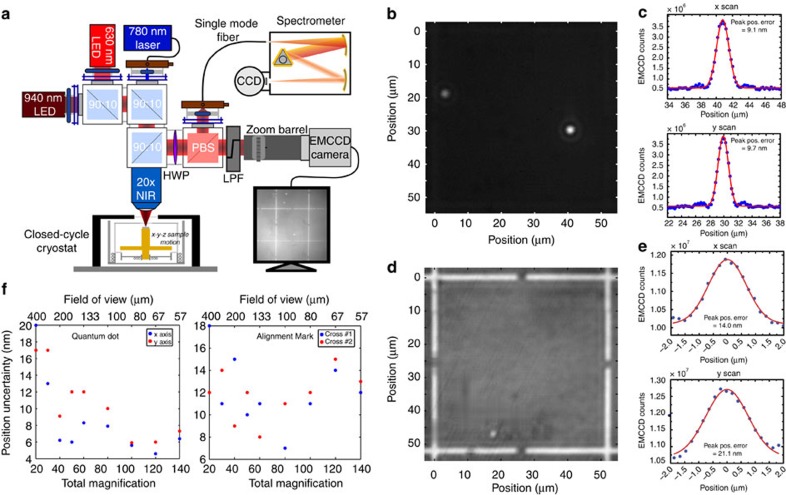
Optically locating single QDs. (**a**) Schematic of the photoluminescence imaging set-up. An infrared LED (emission centred at 940 nm) is used for illumination of the sample while either a 630-nm red LED or a 780-nm laser is used for excitation of the QDs, depending on whether excitation over a broad area (LED) or of individual QDs (laser) is required. Samples are placed within a cryostat on an *x*–*y*–*z* positioner. Imaging is done by directing the emitted and reflected light into an EMCCD camera, while spectroscopy is performed by collecting emission into a single-mode fibre and sending it to a grating spectrometer. (**b**) Example photoluminescence image from single QDs measured under red LED illumination only. A 900-nm LPF is inserted into the collection path when measuring the QD emission. (**c**) Two orthogonal line cuts (horizontal=*x* axis, vertical=*y* axis) of the photoluminescence image, showing the profiles of the QD emission (symbols) and their Gaussian fits (lines). (**d**) Example image of the reflected light from the metallic alignment marks under red LED illumination only. (**e**) Two orthogonal line cuts (horizontal=*x* axis, vertical=*y* axis) of the image in (**d**), showing the profiles of the reflected light from the metallic alignment marks (symbols) and their Gaussian fits (lines). (**f**) Peak position uncertainties measured from the Gaussian fits of line cuts of the EMCCD images, plotted as a function of magnification and field of view for the QD and metallic alignment marks. The uncertainties represent 1 standard deviation values determined by a nonlinear least squares fit of the data.

An array of metal alignment marks is fabricated on quantum-dot-containing material through a standard lift-off process before sample interrogation (see Methods section). The samples are then placed on a stack of piezo-electric stages to allow motion along three orthogonal axes (*x*,*y*,*z*) within a closed-cycle cryostat that reaches temperatures as low as 6 K. The simplest photoluminescence imaging configuration we use is a subset of Fig. 1a, and starts with excitation by a 630-nm light emitting diode (LED), which is sent through a 90/10 (reflection/transmission percentage) beamsplitter and through a 20 × infinity-corrected objective (0.4 numerical aperture) to produce an ≈200 μm diameter spot on the sample. Reflected light and fluorescence from the sample goes back through the 90/10 beamsplitter and is imaged onto an Electron Multiplied Charged Couple Device (EMCCD) using a variable zoom system. When imaging the fluorescence from the QDs, the 630-nm LED power is set to its maximum power (≈40 mW, corresponding to an intensity of ≈130 W cm^−2^), and a 900-nm long-pass filter (LPF) is inserted in front of the EMCCD camera to remove reflected 630 nm light. Imaging of the alignment marks is done by reducing the LED power to 0.8 mW, turning off the EMCCD gain and removing the 900-nm LPF.

Representative images of the QD photoluminescence and alignment marks are shown in Fig. 1b,d. In Fig. 1b, circular bright spots surrounded by Airy rings—a signature of optimally focused collection—are clearly visible and represent the emission from single QDs excited within an ≈56 μm × 56 μm field of view. Orthogonal line scans of the bright spots (Fig. 1c) are fit with Gaussian functions using a nonlinear least squares approach (see Supplementary Note 1), with the extracted peak positions showing one standard deviation uncertainties as low as ≈9 nm. A similar analysis of orthogonal line scans of the alignment marks (Fig. 1d,e) shows their centre positions to be known with an uncertainty that is typically ≈15 nm. Figure 1f shows how this uncertainty changes as a function of system magnification (and hence field of view), which is adjusted using the variable zoom system. We see that the QD uncertainty values show a decreasing trend with higher magnification, and values as low as ≈5 nm are measured. This can be understood because the increased magnification spreads the QD emission over a larger number of pixels on the EMCCD camera, resulting in a smaller fit uncertainty, provided that the collected fluorescence level produces an adequate per pixel signal-to-noise level. On the other hand, the uncertainty in the alignment mark centre position shows no obvious trend with changing magnification. Ultimately, we have found that the alignment mark uncertainties are limited by the blur induced by the two intermediate fused silica cryostat windows (vacuum and radiation shield, 2 mm and 1 mm thick, respectively) between the objective and sample, which has been confirmed by measurements in ambient conditions with the windows removed.

While the 630-nm LED can thus be used for imaging both the QDs and alignment marks, it requires the acquisition of two separate images, with insertion of a filter needed when collecting the QD photoluminescence. As filter insertion can result in beam shifts that will be manifested as an uncontrolled error in determination of the separation between QD and alignment mark, we implement a modified set-up (Fig. 1a) in which a second, infrared LED at 940 nm is combined with the 630-nm LED when illuminating the sample. Unlike the 630-nm LED, the 940-nm LED does not excite the QDs, but instead serves only to illuminate the alignment marks, with the wavelength chosen to approximately match the expected wavelength of the QD emission. By adjusting the 940-nm LED power appropriately, both the QDs and alignment marks can be observed in a single image with the 900-nm LPF in place.

**Figure 2:**
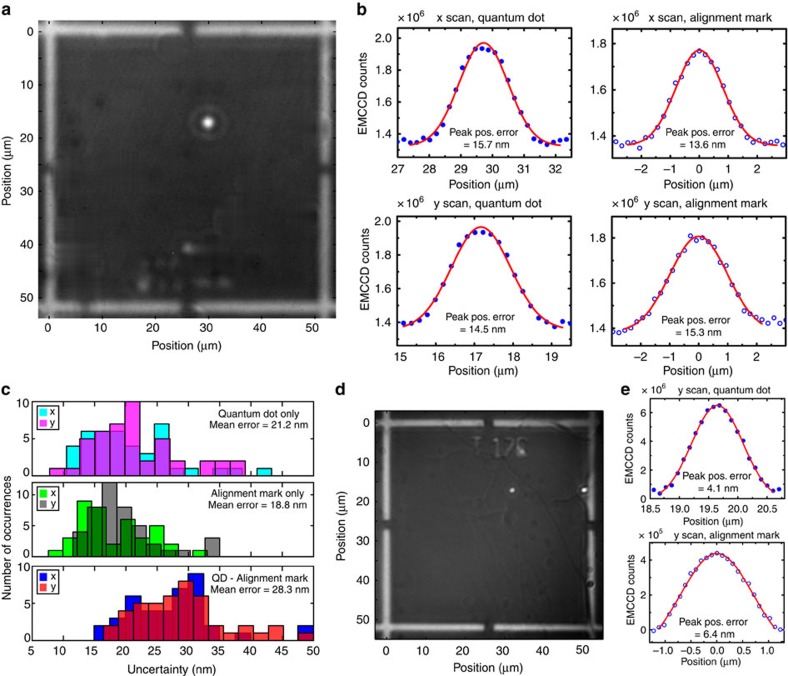
Performance of the two-colour positioning technique. (**a**) EMCCD image of the photoluminescence from a single QD and reflected light by the alignment marks (metallic crosses), acquired by illuminating the sample simultaneously with both the red and near-infrared LEDs. (**b**) Orthogonal line cuts (horizontal=*x* axis, vertical=*y* axis) of the photoluminescence image, showing the profiles of the QD emission (solid symbols) and of the image of the alignment marks (open symbols) and their Gaussian fits (solid lines). (**c**) Histograms of the uncertainties of the QD and alignment mark positions and QD-alignment mark separations, measured from the Gaussian fits of line cuts from 45 images. The uncertainties represent one standard deviation values determined by a nonlinear least squares fit of the data. (**d**,**e**) Photoluminescence imaging through a solid-immersion lens. (**d**) Image of the photoluminescence from single QDs and reflected light from the alignment marks (metallic crosses), collected under the 630 nm/940 nm co-illumination scheme. (**e**) *y* axis line cuts from the photoluminescence image, showing the profiles of the QD emission (solid symbols) and reflected light from the alignment mark (open symbols). The solid lines are nonlinear least squares fits to Gaussians.

Figure 2a shows an image taken when the sample is co-illuminated by both 630 and 940 nm LEDs, with the 940-nm power set to be ≈4 μW, about four orders of magnitude smaller than that of the 630-nm LED power. Orthogonal line scans through the QD and alignment marks under this co-illumination scheme are shown in Fig. 2b. As expected, the uncertainty values determined for QD and alignment mark positions are larger than those obtained when acquiring two separate images (Fig. 1c,e), for which the LED power can be optimized independently to maximize the image contrast and minimize each uncertainty. However, we have favoured the co-illumination approach due to its ability to reduce some potential uncertainties, like sample drift, that may occur during schemes requiring multiple images to be acquired. Ultimately, one might envision time-multiplexing and drift compensation techniques being employed to correct for such factors.

After carrying out a systematic study of the position uncertainties as a function of magnification, integration time, and EMCCD gain, we have found optimized settings for image acquisition (under × 40 magnification), in terms of the combined QD and alignment mark uncertainty: an integration time of 120 s, an EMCCD gain of 200 and the aforementioned LED powers. Under these conditions, we have studied the uncertainties in the QD position, alignment mark position and QD-alignment mark separation for a number of different QDs on our sample. Histograms of the measured values are reported in Fig. 2c, and show that the mean uncertainty in the QD-alignment mark separation is ≈28 nm. Finally, we note that in the present set-up, the available 630 nm LED power is below that required to saturate the QD emission (a comparison with the saturation counts obtained under laser excitation shows that it is about half the value required). Higher 630 nm LED power would increase the collected photoluminescence and reduce the uncertainty values that we have reported. This pre-eminent role of collected photon flux is well-established in the single emitter localization literature^11^. We have confirmed it in our experiments by using a solid-immersion lens^12^,^13^, which can both increase the LED intensity at the QD and the fraction of QD emission that is collected by the microscope objective. Placing a hemispherical lens with refractive index *n*=2 on the surface of the sample yields individual QD and alignment mark position uncertainties of ≈5 nm (Fig. 2d,e), so that the overall uncertainty in locating the QD with respect to the alignment mark is <10 nm (more details provided in Supplementary Note 2). In total, we note that the positioning uncertainties that we obtain are 2 to 5 times smaller than previously reported^6^,^8^,^9^, and are obtained with a single image, acquired over a 120 s acquisition time, and spanning an area of the sample larger than 100 μm × 100 μm.

### Realization of circular Bragg grating bullseye cavities

We now use the optical positioning technique to fabricate nanophotonic structures tailored for the properties of a specific QD and engineered to enhance the collection efficiency of single photons in free space. First, we obtain information about the QD emission wavelength by spatially selecting one QD and collecting its emission into a single-mode fibre that is coupled into a grating spectrometer (a half waveplate and polarizing beamsplitter are used to switch the collection path between the EMCCD camera and single-mode fibre). Spatial selection is achieved by exciting individual QDs with a 780-nm laser, incorporated into the same micro-photoluminescence set-up (Fig. 1a), and producing a focused spot size of ≈2 μm on the sample surface. The half waveplate and polarizing beamsplitter also enable determination of the QD polarization. Having thus obtained emission wavelength to go along with the spatial position obtained from the imaging set-up, a properly calibrated fabrication process can enable the creation of nanophotonic structures that are tailored to the specific emitter properties. This allows one to minimise (and potentially avoid altogether) the need for mutual spectral tuning of the emitter with respect to the optical resonance of the cavity, which is a clear limitation of the scalability of these sources.

**Figure 3:**
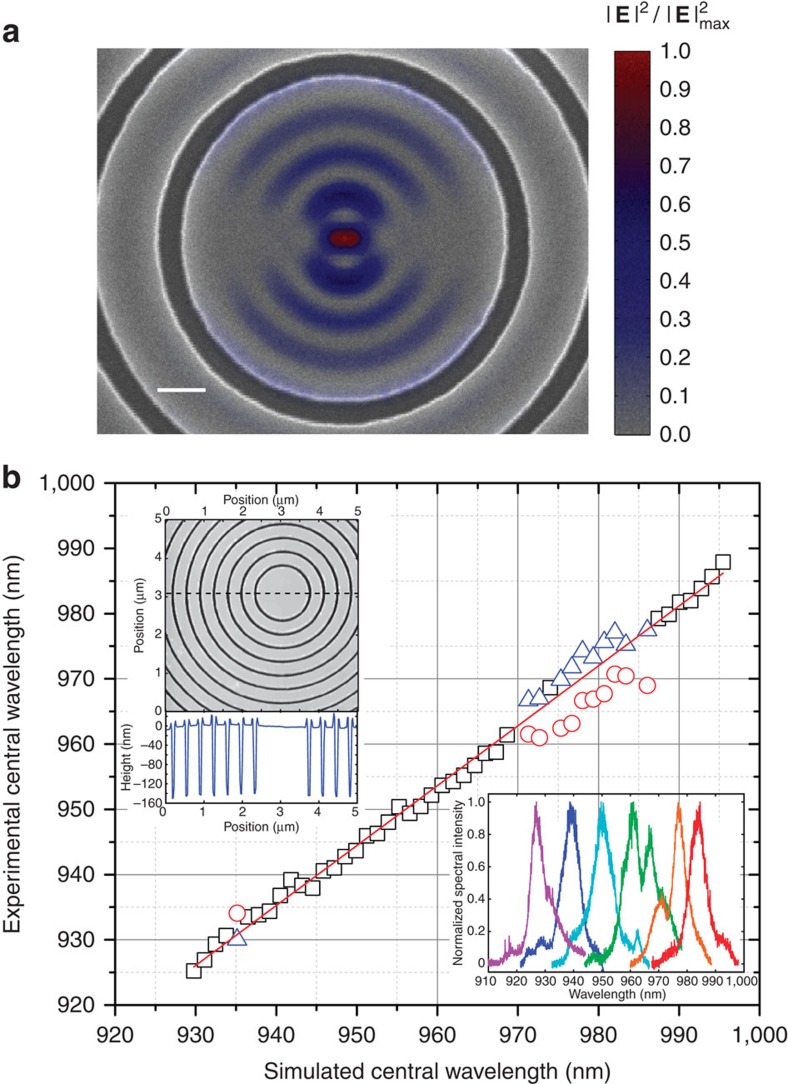
Circular dielectric gratings tailored to specific QD emitters. (**a**) Normalized cavity mode electric field intensity |***E***|^2^ superimposed on a scanning electron microscope image of the centre of one of the cavities. Scale bar represents 200 nm. (**b**) Experimental central wavelength of 50 circular grating cavities with varying period and central radius, plotted as a function of the simulated central wavelength. When only one peak is observed in the spectrum, black squares are used to denote the peak wavelength. When two peaks are observed, red circles and blue triangles are used. Such two-peak behaviour is also seen in simulations depending on the device parameters, and is due to coupling to a second cavity mode. Top inset: Atomic Force Microscope image of a circular grating cavity and a line cut (along the dashed line) showing the etch depth of the trenches. Bottom inset: examples of photoluminescence spectra of circular grating cavities, measured from a high-QD density region.

The specific nanophotonic structure we focus on is a circular Bragg grating ‘bullseye' geometry, which has been developed as a planar structure in which QD photons are funneled into a near-Gaussian far-field pattern over a moderate spectral bandwidth (few nm) with high efficiency (theoretical efficiency of 50% into a 0.4 numerical aperture) and with the potential for Purcell enhancement of the radiative rate^14^,^15^. The cavity mode of interest is tightly confined, and optimal performance requires the QD to be within a couple hundred nanometres of the centre of the bullseye structure. This is illustrated in Fig. 3a, which plots the normalized electric field intensity superimposed on a scanning electron microscope image of the centre of a fabricated device. An important parameter in the fabrication of these devices is the etch depth of the asymmetric grating, as this determines the fraction of emission in the upwards direction (towards our collection optics) compared with the downwards direction (towards the substrate). Furthermore, given the high refractive index difference between GaAs and air, a change in etch depth of 1 nm results in a shift of the optical resonances of about 1 nm. We use AFM to determine the GaAs dry etch rate within the grating grooves (Fig. 3b, top inset), and based on this calibration, we fabricate (see Methods section) 50 circular gratings whose parameters (pitch and central diameter) have been adjusted so that the cavity resonances cover the 930–1,000 nm range of wavelengths. These samples were fabricated in a region of the wafer with a high density of QDs, so that the resulting emission under high power excitation is broad enough to feed the cavity modes. Example spectra collected from different circular grating cavities are shown in the bottom inset of Fig. 3b. These measurements allow us to calibrate the experimental cavity resonances with respect to simulations, as shown in the main panel of Fig. 3b, and tailor the design to match the specific QD emission wavelength.

### Optimized QD single-photon source

**Figure 4:**
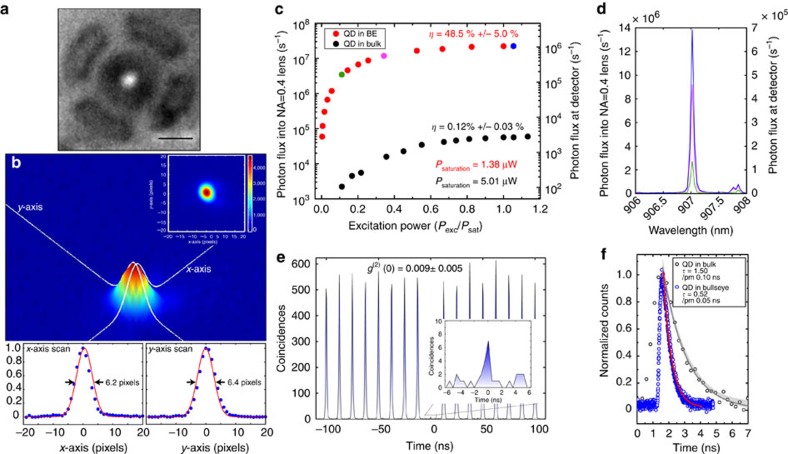
Single-photon emission from an optimised device. (**a**) Image of the photoluminescence from a single QD within the cavity, collected under 630 nm LED illumination. Scale bar represents 5 μm. (**b**) Far-field image of the photoluminescence from a QD in a circular grating cavity, along with line cuts from the two-dimensional Gaussian fit to the data along the *x* and *y* axes, shown as solid white lines. The upper right inset shows a two-dimensional image plot of the interpolated data, while the bottom curves plot the (uninterpolated) experimental data (symbols) and their Gaussian fits (solid lines). (**c**) Photon flux into the 0.4 numerical aperture collection objective (left *y* axis) and at the detector (right *y* axis), plotted as a function of 780 nm excitation power (in saturation units), for a QD in a circular grating (QD in BE, red symbols) and in unpatterned GaAs (QD in bulk, black symbols). (**d**) Examples of photoluminescence spectra collected under different excitation power (colour coded in panel (**c**)). (**e**) Photon collection coincidence events measured under pulsed 857 nm excitation, using a Hanbury–Brown and Twiss set-up. The disappearance of the central peak (zoomed-in plot in the inset) is the signature of pure single-photon emission. The uncertainty value is given by the standard deviation in the area of the peaks away from time zero. See Supplementary Fig. 2 for additional relevant data. (**f**) Time-resolved photoluminescence measurements collected under pulsed 780 nm excitation, showing the excited state decays (symbols) fitted by single exponential curves (solid lines). The shaded grey areas correspond to the 95% confidence intervals in the fit.

Using the QD positions with respect to alignment marks as determined by photoluminescence imaging, emission wavelengths as determined by grating spectrometer measurements, and the aforementioned calibration of the circular grating geometry to match target wavelengths, we fabricate (see Methods section) a series of circular grating cavities containing single QDs. Photoluminescence imaging of the devices after fabrication, as shown in Fig. 4a for a representative device excited by the 630-nm LED, qualitatively indicates that the QD emission originates from the centre of the bullseye structure, as intended. A measurement of the far-field emission from the device on the EMCCD, as shown in Fig. 4b, shows that it is close to a circular Gaussian function, as confirmed by a nonlinear least squares fit. As the overlap with a perfect circular Gaussian is ≈70%, this far-field patten is expected to mode match well to a single-mode fibre, an important consideration for long-distance transmission of single photons for quantum information applications.

We now characterise the emission produced by the optically positioned QDs within the circular grating cavities, in terms of collection efficiency, single-photon purity and spontaneous emission rate. For these measurements, a second cryostat and photoluminescence set-up was used, as it provides direct free-space in-coupling to a grating spectrometer that is also used for spectral isolation of the QD excitonic state (Supplementary Fig. 1). First, we determine the collection efficiency by pumping the devices with a 780 nm wavelength, 50 MHz repetition rate pulsed laser (50 ps pulse width), and varying the laser power until the emission from the QD saturates (Fig. 4c). Assuming a QD radiative efficiency of unity, and taking into account the losses within the optical set-up (see Supplementary Note 3), we measure a collection efficiency as high as 48.5%±5.0% into a 0.4 numerical aperture objective, where the uncertainty is due to fluctuations in power measurements done to calibrate losses in the optical set-up, and represents the value of one standard deviation. This collection efficiency is close to the theoretical value of 50% expected for a centrally located QD, and is more than two orders of magnitude larger than the collection efficiency for a QD in unpatterned GaAs, as shown in Fig. 4c. We note that a 80% collection efficiency is theoretically expected if a higher numerical aperture optic (for example NA=0.7) is used.

In previous studies of QDs in circular grating cavities^14^,^15^, where no optical positioning was used, device fabrication in a material containing a higher density of QDs was performed, to ensure that some non-negligible fraction (which turned out to be a few per cent) of devices would have a QD spectrally and spatially overlapped with the desired cavity mode (see Supplementary Note 4 and Supplementary Figure 3). In comparison, the optical positioning used here allows us to work with a much lower density of QDs (≲1 per 1,000 μm^2^). One consequence of this is the comparatively clean emission spectra we observe, even when exciting with pump powers that completely saturate the QD emission (Fig. 4d). Such clean spectra might be expected to correspond to clean (low multiphoton probability) single-photon emission, and to test this, the spectrally filtered emission from the bright QD exciton line is measured in a standard Hanbury–Brown and Twiss set-up. Under non-resonant, 780 nm pulsed excitation, we measure *g*^(2)^(0)=0.15±0.03 when the QD emission is saturated (Supplementary Fig. 2a). When the system is excited above QD saturation non-resonantly, we observe emission from the bullseye cavity modes superimposed with the QD emission (data in Supplementary Fig. 2b, collected under continuous wave 780 nm excitation). Together, this suggests that quasi-continuum states, originating from the combined single QD—wetting layer system, feed the optical cavity mode^16^,^17^,^18^ and limit the device's single-photon purity.

We next consider pumping the device on an excited state of the QD, as such excitation (sometimes referred to as quasi-resonant or p-shell pumping) has been shown to reduce *g*^(2)^(0) (ref. 19). Measurement of the QD emission under pulsed 857 nm excitation shows that, at the saturation pump intensity (where the collection efficiency is maximized), the spectrum is nearly identical to that under 780 nm excitation (Supplementary Fig. 2d). Moreover, increased excitation power above saturation (achieved using a 857 nm continuous wave laser) yields far less cavity mode feeding than in the corresponding 780 nm case (Supplementary Fig. 2c), suggesting that improved single-photon purity should be observed. This is confirmed by intensity autocorrelation measurements, which indicate that on-demand single-photon emission with a purity of 99.1% (*g*^(2)^(0)=0.009±0.005) is achieved at QD saturation. We note that the *g*^(2)^(0) levels are determined from raw coincidences, without any background subtraction, and with an uncertainty value given by the standard deviation in the area of the peaks away from time zero.

We also measure the spontaneous decay rate of the QD emission under 780 nm pulsed excitation (measurements at 857 nm have also been performed and yield unchanged results). The spontaneous emission decay of a QD in bulk and a QD in a circular grating cavity are shown in Fig. 4f. The exponential fit of the decay curve allows us to extract a lifetime of ≈520 ps for the QD in the bullseye cavity, corresponding to a Purcell enhancement of the spontaneous emission rate by a factor of ≈3. A Purcell factor as high as 4 is measured in other devices that have a smaller detuning with respect to the cavity mode (the detuning is 1.6 nm for the device we focus on here). Theoretically, Purcell factors as high as ≈11 are expected^14^,^15^ for QDs with perfect spectral and spatial alignment with respect to the cavity mode. Different methods to achieve such precise spectral resonance are currently under consideration; preliminary measurements indicate that *in situ* N_2_ deposition is ill-suited to the circular grating geometry, as the cavity mode degrades before a significant wavelength shift is observed.

Going forward, it would be relevant to determine the location of the optically positioned QDs within fabricated devices, in order to understand sources of error within our overall fabrication approach (which combines optical positioning with aligned electron-beam lithography). Supplementary Note 5 and Supplementary Figures 4–9 present a detailed discussion on the results of finite-difference time-domain simulations examining the Purcell factor, collection efficiency, and degree of polarization in the collected far-field as a function of dipole position and orientation within the cavity. Our calculations indicate that the Purcell enhancement, in particular, very sensitively depends on the dipole location, while the collection efficiency is not as sensitive (see Supplementary Figures 6 and 7). For the devices we have focused on in the main text, we find that a simulated offset between 50 and 250 nm with respect to the cavity centre produces results that are consistent with our measurements.

## Discussion

There has been much progress in the development of bright QD single-photon sources in recent years, including micropillar^20^,^21^, vertical nanowire waveguide^22^,^23^,^24^, fibre-coupled microdisk^25^ and photonic crystal cavity^26^ geometries. Many metrics are needed to characterize these sources, and the choice of which ones are of particular importance is largely determined by the intended application. Within the landscape of these sources, the results presented here are unique in terms of simultaneously exhibiting high collection efficiency, nearly perfect single-photon purity at the highest measured collection efficiency, and Purcell enhancement of the spontaneous emission rate. For example, previous bright, Purcell-enhanced microcavity single-photon sources have shown significant non-zero *g*^(2)^(0) values (≳0.1) at their highest collection efficiencies^20^,^21^,^25^,^26^, while bright nanowire sources show *g*^(2)^(0)≈0 but do not exhibit Purcell enhancement^22^,^23^,^24^. For some applications, the metrics demonstrated thus far should be combined with a high degree of photon indistinguishability^21^, which is limited in our work by the coherence time of the QDs in this sample (<300 ps, as confirmed by measurements with a scanning Fabry Perot interferometer; other emitters on the same wafer show coherence times as long as 500 ps). Future work will focus on resonant excitation^27^,^28^,^29^ to improve the coherence time and fine control of the cavity-QD detuning to achieve shorter radiative lifetimes^30^,^31^. Together, these advances may provide a route to a source that simultaneously provides bright, pure and indistinguishable single photons.

In conclusion, we have developed a photoluminescence imaging technique that enables the location of single QDs with respect to alignment markers with an average position uncertainty <30 nm and reaching values as low as <10 nm. We have combined this technique with systematic calibration of our fabrication process to create single-photon sources based on a circular Bragg grating geometry that simultaneously exhibit high brightness, purity and Purcell enhancement of the spontaneous emission rate. More generally, this technique is an important step forward in the ability to create functional single QD nanodevices, including quantum light sources, strongly-coupled QD—microcavity systems for achieving single-photon nonlinearities^32^,^33^,^34^, coupled QD—nanomechanical structures^35^,^36^,^37^, and integrated systems involving multiple QD nodes.

## Methods

### Circular Bragg grating cavity fabrication

Devices are fabricated in a wafer grown by molecular beam epitaxy, consisting of a single layer of InAs QDs embedded in a 190-nm thick layer of GaAs, which in turn is grown on top of a 1-μm thick layer of Al_*x*_Ga_1−*x*_As with an average *x*=0.65. The s-shell peak of the QD ensemble is located near 940 nm, and a gradient in the QD density is grown along one axis of the wafer. Low-temperature photoluminescence imaging of portions of the wafer is performed before any device definition to determine the appropriate location on the wafer (in terms of QD density) to fabricate devices.

Alignment marks are fabricated using positive tone electron-beam lithography and a lift-off process. Polymethyl methacrylate with a molecular weight of 495,000 is spin coated onto the sample, and 2 μm wide, 50 μm long crosses are patterned in the resist using a 100-keV electron-beam lithography tool. After exposure, the resist is developed in a 1:3 (by volume) solution of methyl isobutyl ketone and isopropanol, and 20 nm of Cr and 100 nm of Au are deposited on the sample using an electron-beam evaporator. Microposit remover 1165 is used for lift-off, with gentle ultra-sonication applied if necessary.

After location of QDs with respect to the alignment marks through photoluminescence imaging, circular Bragg grating ‘bullseye' microcavities are fabricated as follows. First, the sample is spin coated with a positive tone electron-beam resist (ZEP 520A), and aligned electron-beam lithography with a 100-keV tool and four mark detection is performed. Next, the pattern is transferred into the GaAs layer using an Ar–Cl_2_ inductively-coupled plasma reactive ion etch. After removal of the electron-beam resist, the sample is undercut in hydrofluoric acid.

AFM was used in the calibration of the etch rate, with the samples scanned in tapping mode using a commercial, etched silicon probe whose backside is coated with Al. The AFM probe cantilever has a vendor-specified spring constant of 42 N/m, frequency of 300 kHz, and probe tip radius and height of 8 nm and 10 μm, respectively.

## Additional information

**How to cite this article:** Sapienza, L. *et al*. Nanoscale optical positioning of single quantum dots for bright and pure single-photon emission. *Nat. Commun*. 6:7833 doi: 10.1038/ncomms8833 (2015).

## Supplementary Material

Supplementary Information Supplementary Figures 1-9, Supplementary Notes 1-5 and Supplementary References
