# Supplementary material for: Nanoscale optical positioning of single quantum dots for bright and pure single-photon emission
Source: Nat Commun. 2015 Jul 27;6:7833. doi: 10.1038/ncomms8833 (PMC4525159; doi:10.1038/ncomms8833)
Supplement: Supplementary Information — Supplementary Figures 1-9, Supplementary Notes 1-5 and Supplementary References [file ncomms8833-s1.pdf]

Supplementary Figures

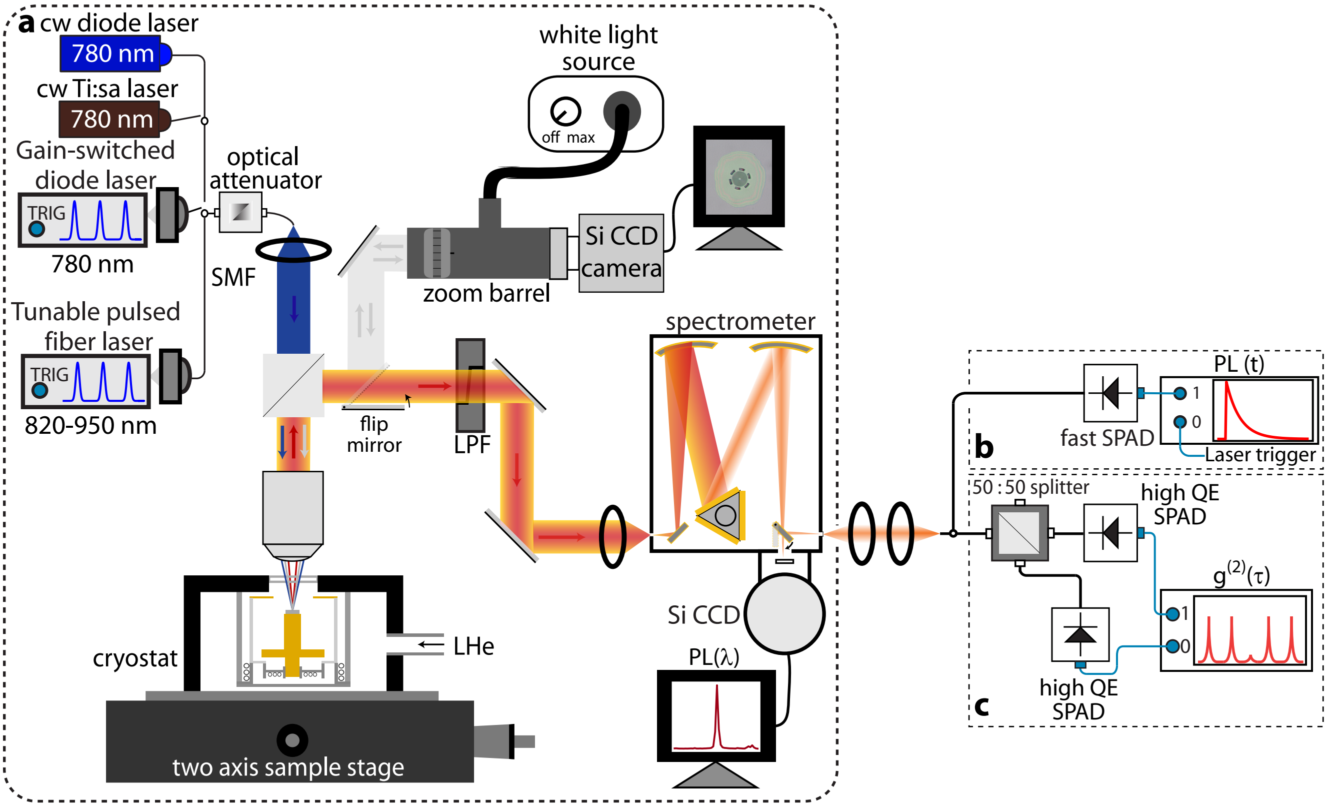

Supplementary Figure 1: Schematic of the experimental setup: (a) confocal micro-photoluminescence; (b) time-resolved photoluminescence; (c) Hanbury Brown and Twiss photon correlation setup. SMF: single-mode fiber, LPF: long-pass filter, CCD: charge-coupled device, PL: photoluminescence, SPAD: single-photon avalanche diode, QE: quantum efficiency.

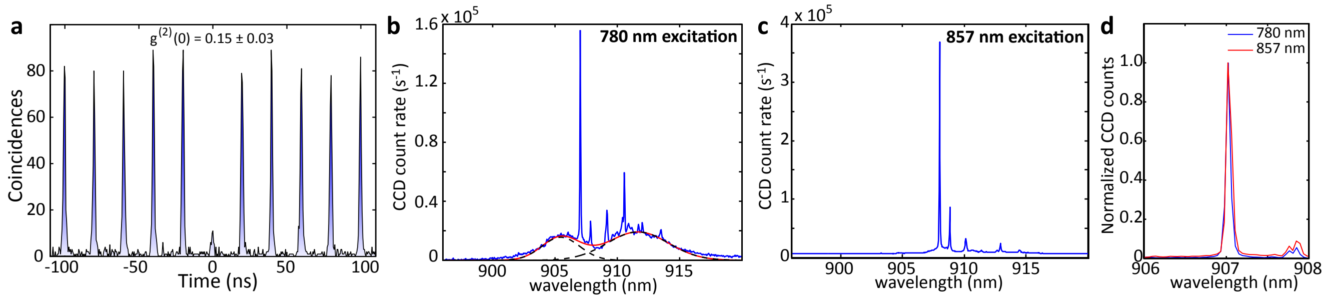

Supplementary Figure 2: Supplementary data for the single photon source characterization of Figure 4 from the main text. (a) $g^{(2)}(\tau)$ under 780 nm pulsed excitation, with the quantum dot emission saturated. (b) Spectrum under intense 780 nm continuous wave excitation (far above quantum dot saturation), for which the bullseye cavity modes are visible. The quantum dot line of interest is +1.6 nm detuned with respect to the shorter wavelength cavity mode. The red solid line is a fit of the data to the sum of two Gaussians, which is used to determine the center wavelengths of the two cavity modes. The two Gaussians making up the sum are shown as black dashed lines. (c) Spectrum under intense 857 nm continuous wave excitation (far above quantum dot saturation), on resonance with a quantum dot excited state. Compared to 780 nm excitation far above saturation, significantly reduced cavity mode emission is observed. (d) Comparison of the quantum dot spectra under saturation conditions (the conditions under which the $g^{(2)}(\tau)$ data in part (a) and Fig. 4(e) were taken) for both 780 nm and 857 nm pulsed excitation.

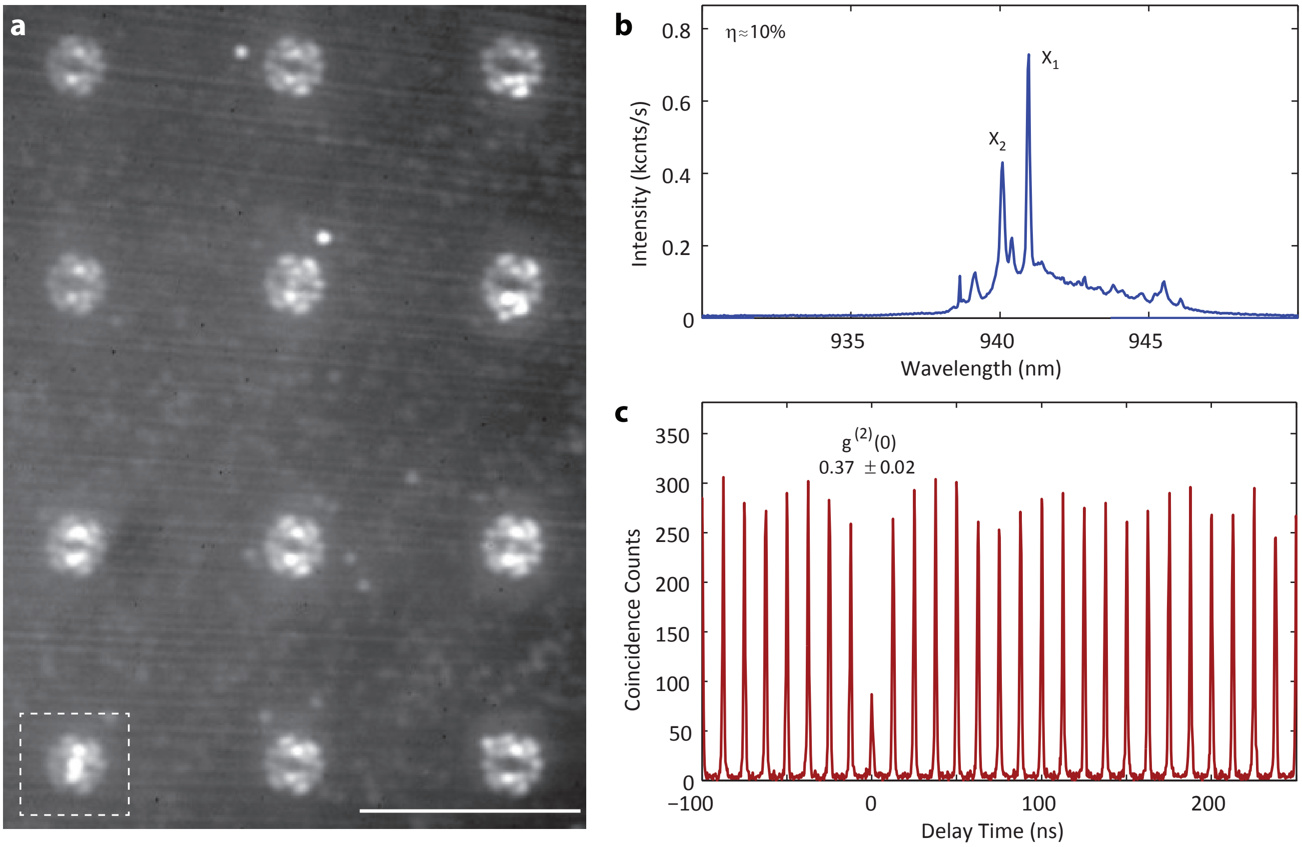

Supplementary Figure 3: Representative data from quantum dot - bullseye cavity devices fabricated without using the quantum dot positioning technique, as in Ref. 2. (a) EMCCD image of an array of cavities, illuminated by a 630 nm LED. Only one of the devices (within the dashed box) shows an emission lobe centered with respect to the cavity. Scale bar represents 50 μm. (b) Photoluminescence spectrum from a device exhibiting collection efficiency $\approx$ 10 %. (c) $g^{(2)}(\tau)$ from the same device. Note that parts (b) and (c) are re-displayed data from Ref. 2.

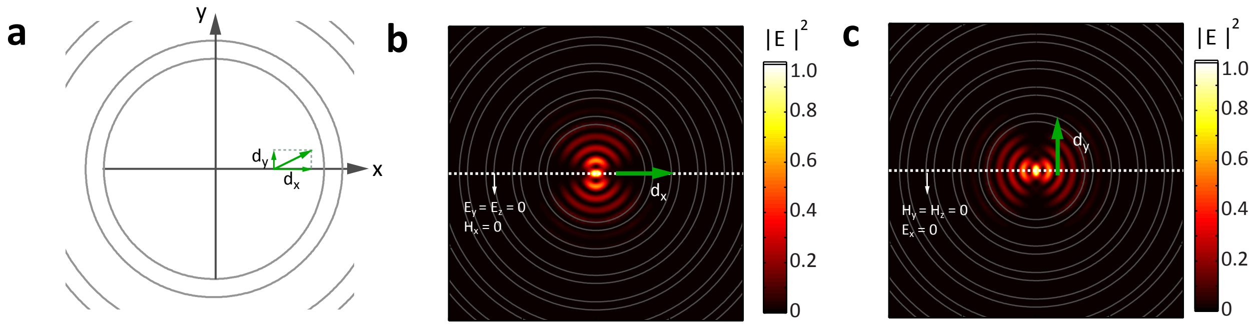

Supplementary Figure 4: (a) Schematic of the simulation geometry, showing a dipole (green arrows) inside a bullseye cavity. Due to the circular symmetry, a dipole located anywhere within the cavity and with any orientation can be represented by a dipole on the x-axis with d_x and d_y components equivalent to the radial and azimuthal ones. (b) Electric field amplitude squared profile for a 'h'-type bullseye cavity mode. The yz-plane boundary conditions satisfied by the mode are given in the figure. This mode is only excited by x-dipoles. (c) Electric field amplitude squared profile for a 'v'-type bullseye cavity mode. The yz-plane boundary conditions satisfied by the mode are given in the figure. This mode is only excited by y-dipoles.

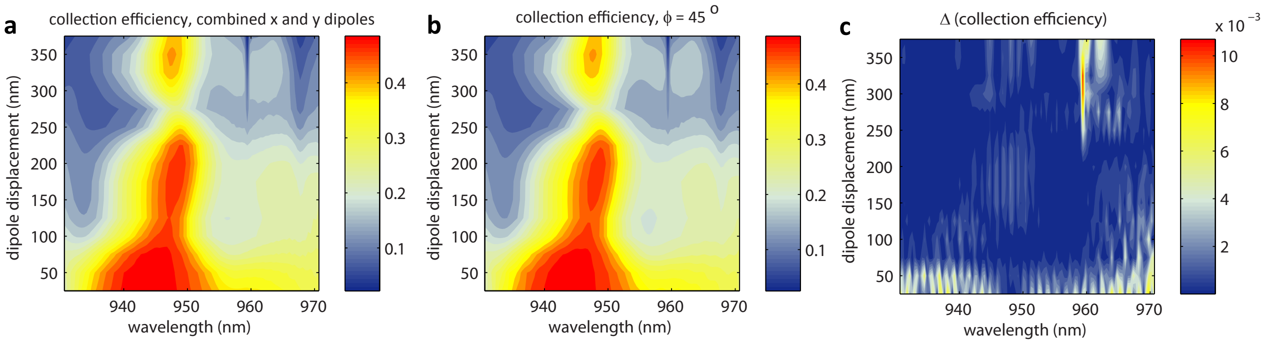

Supplementary Figure 5: Collection efficiency $\eta_{0.4}$ into a $NA = 0.4$ objective as a function of wavelength and dipole position along the x-axis inside the bullseye cavity. The dipole is on the xy-plane and is oriented at an azimuthal angle $\phi = 45°$. (a) Results obtained using eq.(1) with separate simulations for x and y dipoles individually. (b) Results obtained by simulating a dipole at $\phi = 45°$. (c) Absolute value of difference between results shown in panels (a) and (b).

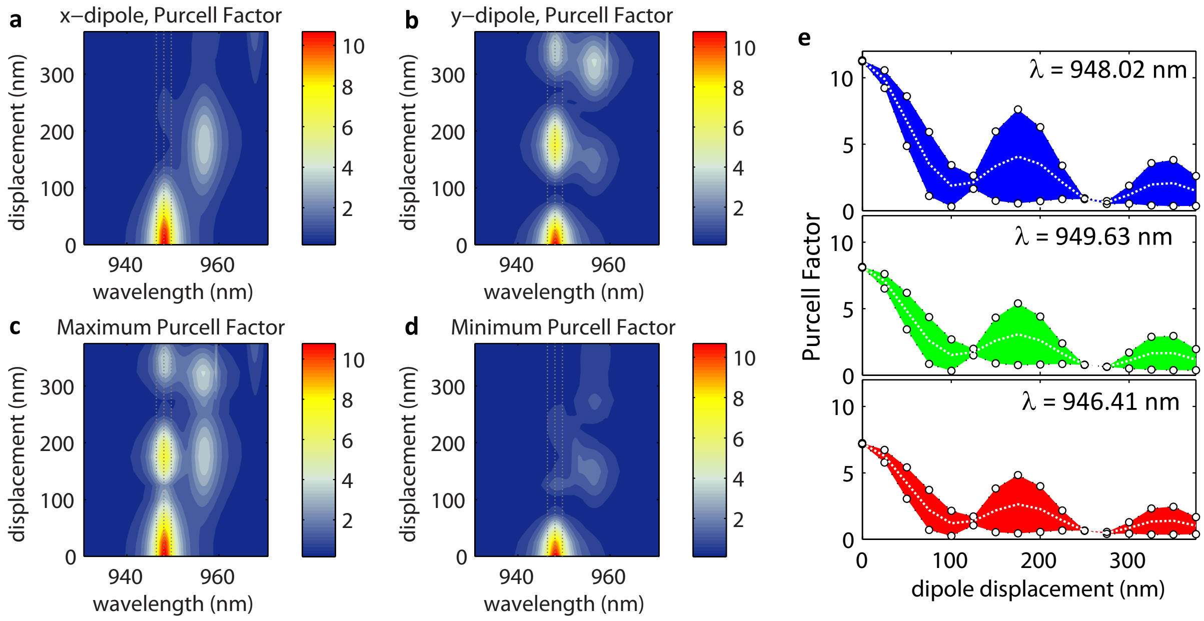

Supplementary Figure 6: Purcell Factor F_p as a function of wavelength and dipole position along the x-axis, inside the bullseye cavity. (a) Results for an x-oriented dipole; (b) Results for a y-oriented dipole; (c) maximum achievable F_p; (d) minimum achievable F_p; (e) Purcell Factor as a function of dipole displacement from the bullseye cavity center, at resonance ($\lambda = 948.02$ nm) and at $\pm$ 1.6 nm away [shown as dashed lines in (a)-(d)]. Shaded areas correspond to the uncertainty in F_p due to lack of knowledge of the dipole azimuthal angle ϕ. The white dotted line corresponds to the case $\phi = 45°$.

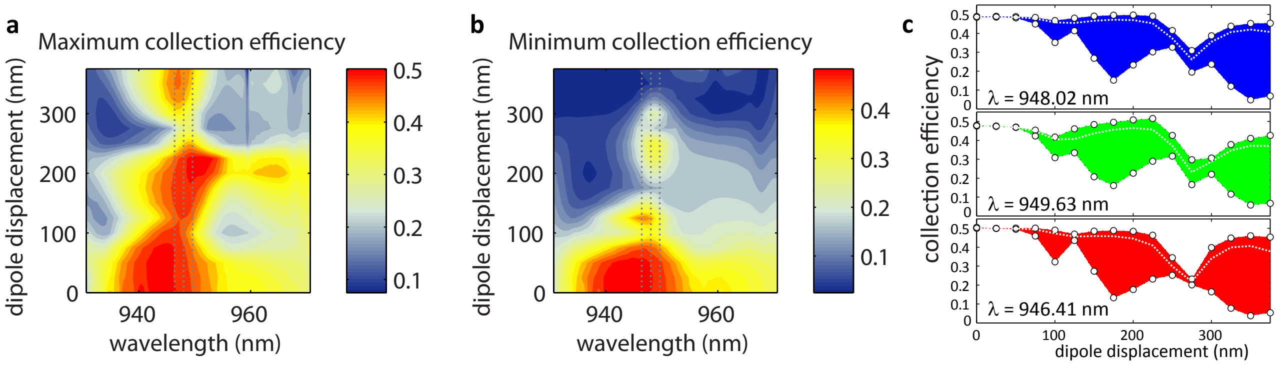

Supplementary Figure 7: Collection efficiency $\eta_{0.4}$ into a $NA = 0.4$ optic as a function of wavelength and dipole position along the x-axis inside the bullseye cavity. (a) Maximum achievable $\eta_{0.4}$; (b) minimum achievable $\eta_{0.4}$; (c) Collection efficiency as a function of dipole displacement from the bullseye cavity center, at resonance ($\lambda = 948.02$ nm) and $\pm$ 1.6 nm away [shown as dashed lines in (a) and (b)]. Shaded areas correspond to the uncertainty in $\eta_{0.4}$ due to lack of knowledge of the dipole azimuthal angle ϕ. The white dotted line corresponds to the case $\phi = 45°$.

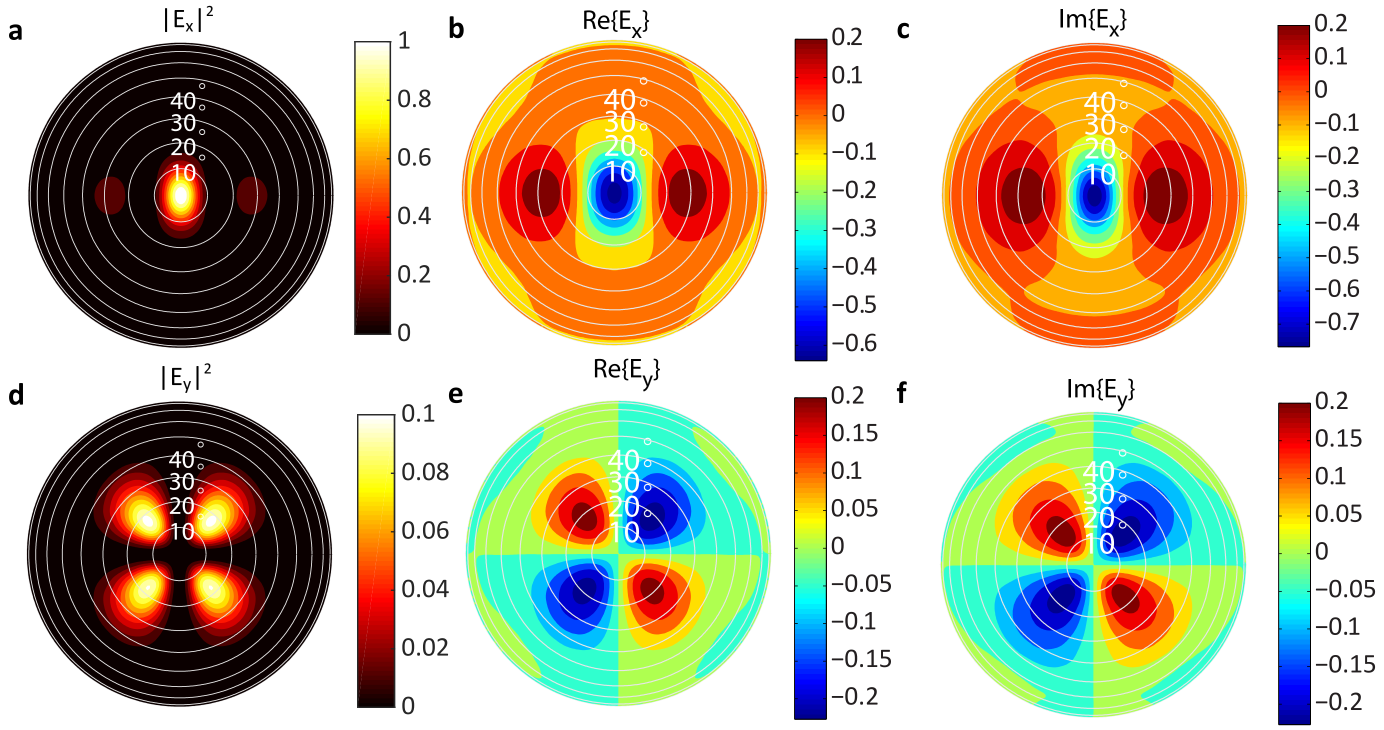

Supplementary Figure 8: (a) Magnitude-square, (b) Real part, and (c) Imaginary part of the E_x far-field for a 'v' mode. d) Magnitude-square, (e) Real part, and (f) Imaginary part of the E_y far-field for a 'v' mode.

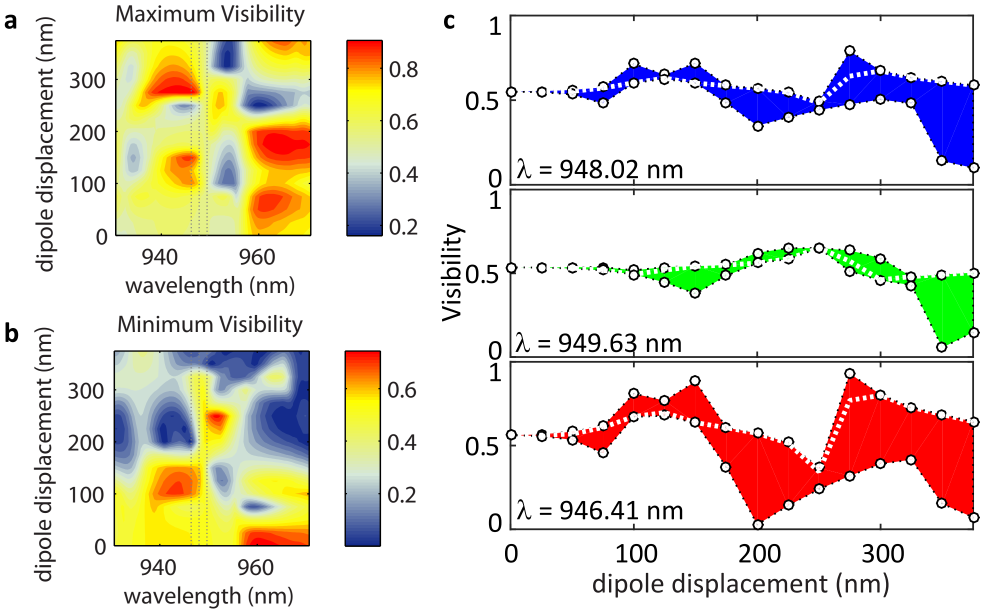

Supplementary Figure 9: Visibility V as a function of wavelength and dipole position along the x-axis inside the bullseye cavity. (a) Maximum V; (b) minimum V; (c) Visibility as a function of dipole displacement from the bullseye cavity center, at resonance ($\lambda = 948.02$ nm) and $\pm$ 1.6 nm away [shown as dashed lines in (a) and (b)]. Shaded areas correspond to the uncertainty in V due to lack of knowledge of the dipole azimuthal angle ϕ. The white dotted line corresponds to the case $\phi = 45^{\circ}$.

Supplementary Note 1: Quantum dot positioning setup and measurements

In this note, we provide additional details on the quantum dot positioning setup shown schematically in Fig. 1 of the main text.

The samples are housed within a cryogen-free cryostat with a base temperature as low as 6 K. Sample motion is achieved using a three-axis cryogenic piezo-positioning stage system. A confocal micro-photoluminescence geometry is utilized, in which a microscope objective (20x magnification and 0.4 numerical aperture) both focuses excitation light on the sample and collects light emitted and reflected by the sample. As described in the main text, photoluminescence imaging is done with co-illumination by 630 nm and 940 nm LEDs, where the former is used to excite the quantum dots and the latter is used to image alignment marks. Excitation of single quantum dots for spectroscopy is performed by focusing a 780 nm laser on the sample.

A 90/10 (reflection/transmission percentage) beamsplitter followed by a 900 nm long-pass filter is used to send the light emitted and reflected by the sample towards the imaging and spectroscopic characterisation paths. Selection between the two paths is accomplished with a half waveplate and polarizing beasmplitter. For photoluminescence imaging, the collected light is coupled into a variable zoom system and Electron Multiplied Charged Couple Device (EMCCD), while for spectroscopy, it is coupled to a single mode fiber whose output is sent to a grating spectrometer equipped with a silicon Charged Coupled Device (CCD).

In the photoluminescence imaging measurements, the 900 nm longpass filter serves to reject 630 nm excitation light, while allowing both the quantum dot emission and reflected 940 nm LED light to pass. A total system magnification of 40x (20x from the objective, and 2x from the zoom barrel) is used, corresponding to a field of view of $\approx$ 200 μm x 200 μm. EMCCD images are acquired with an integration time of 120 s and gain of 200. While the 630 nm LED power is always set at its maximum ($\approx$ 40 mW, corresponding to an intensity of $\approx$ 130 W/cm^2) to generate as much fluorescence from the quantum dot as possible, the 940 nm LED power is set to achieve a reflected signal from the alignment marks that is approximately equal to the intensity of the quantum dot emission. This choice of 940 nm LED power is a tradeoff between the improved alignment mark position uncertainty produced at higher powers, and the degraded quantum dot position uncertainty that results if the reflected 940 nm LED signal swamps the quantum dot emission. A typical 940 nm LED power is $\approx$ 4 μW.

The linecuts of the images taken by the EMCCD camera are analyzed using a commercial software and fitted by Gaussian functions to determine the location of the quantum dot and centers of the alignment marks. The fit is optimized using a Levenberg Marquardt iteration algorithm. The central position of the Gaussian function and its error are then translated from a pixel value on the camera to a distance on the sample by using a calibration obtained by imaging, under the same magnification conditions, a microscope calibration target presenting etched features with known separations.

Supplementary Note 2: Solid immersion lenses for reduced positioning uncertainties

Solid immersion lenses have been used to increase the collection of light emitted by semiconductor quantum dots by increasing the effective numerical aperture of the collection optics[1]. Moreover, because the solid immersion lens reduces the focused excitation spot size, it also increases the excitation intensity at the sample. This can lead to an increased photon flux from the quantum dot, because (as was noted in the main text), without the solid immersion lens, the maximum 630 nm LED excitation intensity at the sample is not enough to saturate the quantum dot emission. Taken together, the increased emission signal from the quantum dot should lead to a lower uncertainty in its position. We also expect that the solid immersion lens can improve the alignment mark uncertainty, since the amount of 940 nm LED power used to image the mark will be increased (see discussion above) to match the increased quantum dot emission level.

We test the above experimentally using a 2 mm diameter, high refractive index ($n \approx 2$) half-ball lens placed directly on the sample surface, with a thin layer of cryogenic grease applied between the sample and lens, obtaining the results shown in Fig. 2(d)-(e) from the main text (the x-axis scans are similar). We measure uncertainties in the quantum dot-marker distance as low as 7.6 nm, a reduction of about a factor 4 compared to the average error measured without the lens (and a factor of 2 compared to the best error measured without the lens).

However, there are some considerations to take into account other than the reduced positioning error possible with a solid immersion lens. First, the solid immersion lenses are generally 1 mm or 2 mm in diameter. Therefore, when using them for imaging the quantum dot emission and alignment marks, the area of the sample that can be probed within a single measurement session is highly reduced, unless multiple lenses are used. Second, given that the lenses are hemispherical, care must be taken in optimally focusing the imaging and excitation light on the apex of the solid immersion lens, in order to avoid distortions of the image that would affect the inferred distance between the alignment mark and the quantum dot.

Supplementary Note 3: Quantum dot single-photon source characterization

A schematic of the experimental setup used to evaluate the collection efficiency and single-photon purity of the quantum dot emission is shown in Supplementary Fig. 1, and is similar to that used in previous work[2]. The sample is mounted on the cold finger of a liquid helium flow cryostat that sits on a two-axis nano-positioning stage. Spectral properties of the quantum dot emission are investigated via low-temperature micro-photoluminescence, where a 20x microscope objective (numerical aperture of 0.4) is used for both the illumination of the sample and the collection of the emission. Four different excitation sources are available for use. The first is a continuous wave 780 nm diode laser for basic spectroscopy. The second is a continuous wave Ti:sapphire laser, tunable beteween 780 nm and 1000 nm, that can be used to excite the quantum dot on its different excited state transitions. The third is a gain-switched, 780 nm pulsed laser diode (50 ps pulse width; 50 MHz repetition rate) for photon counting, lifetime, and correlation measurements. The final source is a 820 nm to 950 nm tunable fiber laser (< 10 ps pulse width; 80 MHz repetition rate) used for counting, lifetime, and correlation measurements under excitation of a quantum dot's excited state.

The collected signal is directed to a spectrometer either to record an emission spectrum with a Si CCD camera, or to filter a single emission line for further investigation (Supplementary Fig. 1(a)). The spectrally filtered emission line is coupled into a single mode optical fiber to enable measurements using fiber-coupled single-photon avalanche diodes (SPADs). Single quantum dot fluorescence decay dynamics are measured through time-correlated single-photon counting, which relies on measuring the time delay between an excitation pulse and detection of an emitted photon by a SPAD (Supplementary Fig. 1(b)). We use a thin Si SPAD whose timing jitter is < 50 ps to enable measurement of fast quantum dot decay dynamics. For the second-order correlation function $g^{(2)}(\tau)$ measurements, the spectrally filtered emission is directed to a Hanbury-Brown and Twiss interferometer that consists of a fiber-coupled, 50/50 non-polarizing beam-splitter and two fiber-coupled single-photon avalanche diodes (SPADs), as shown in Supplementary Fig. 1(c). These SPADs have a timing jitter of $\approx$ 700 ps, and their outputs are connected to a time-correlated single-photon counting board. A time bin width of 512 ps is chosen for the $g^{(2)}(\tau)$ measurements.

Calibration of the quantum dot single-photon source collection efficiency into the 20x (0.4 numerical aperture) objective proceeds as follows. First, the transmission of the optical path from the QD source to the detector is determined. The emitted light escapes the cryostat by traveling through two fused silica windows (total transmission $\approx$ 87 %), it is then collected by a microscope objective (transmission of $\approx$ 70 %), goes through a 90/10 beamsplitter (transmission of $\approx$ 89 %), reflects off four dielectric mirrors and travels through a polarizer (total transmission of $\approx$ 78 %) before being focused through the slit of the grating spectrometer. The total transmission of the optical path up to the spectrometer is 42 % $\pm$ 4 %, where the uncertainty is based on the spread of transmission values measured for the optical components, and represents a one standard deviation value.

Next, a known laser power (22.6 μW) is sent into the spectrometer after being attenuated by an independently measured attenuation (64.21 dB) using a variable attenuator, and the detected counts on the Si CCD coupled to the spectrometer are recorded. The counts measured from a quantum dot, excited with a 50 MHz repetition rate source, are then recorded and compared to the laser counts (taking into account the transmission of the optical path) in order to extract the emitter's single-photon collection efficiency.

Figure 4 and the accompanying discussion in the main text present data characterizing single-photon source performance for an optically positioned quantum dot within a circular grating 'bullseye' cavity. Here, we present supplementary data referred to in the main text discussion. Supplementary Fig. 2(a) shows an intensity autocorrelation measurement ($g^{(2)}(\tau)$) under pulsed excitation at 780 nm when the quantum dot emission is saturated. Despite what appears to be a relatively clean emission spectrum (in terms of an absence of spectral features other than the quantum dot emission) in Fig. 4(d) of the main text, the relatively significant multi-photon component measured ($g^{(2)}(0) = 0.15 \pm 0.03$) indicates the presence of emission that is spectrally resonant with the emission from the quantum dot excitonic line. Quasi-continuum states, generated by hybridization of states of the single quantum dot with those of the wetting layer[3–5], are thought to be a potential source of such multi-photon emission, particularly in Purcell-enhanced (e.g., microcavity) geometries. This is consistent with our measurements, as more intense excitation (through a 780 nm continuous wave laser that provides more output power than the pulsed 780 nm laser) yields a spectrum in which the cavity mode emission is clearly visible (Supplementary Fig. 2(b)). Given that the cavity mode linewidths are many nanometers wide, while the quantum dot excitonic states are orders of magnitude narrower, a spectrally broad emission source such as a quasi-continuum state is needed to reconcile the presence of the cavity mode within the measured spectrum.

The contribution of such quasi-continuum states should be limited if the system is pumped on an excited state of the quantum dot, which would prevent the generation of high energy carriers that could fill those states. Using a narrow linewidth (< 1 MHz) continuous wave Ti:sapphire laser, we have identified 857.0 nm and 876.4 nm as wavelengths that are resonant with quantum dot excited states. Under intense excitation at these wavelengths, the cavity mode feeding is significantly reduced relative to the 780 nm case, as shown in Supplementary Fig. 2(c) for 857 nm excitation. Switching to pulsed 857 nm excitation, we find that the resulting spectrum at saturation of the quantum dot emission is nearly identical to that observed under pulsed 780 nm excitation in the main text, as shown in Supplementary Fig. 2(d). In contrast, the measured $g^{(2)}(\tau)$ (Fig. 4(e) in the main text) is markedly different, with $g^{(2)}(0) = 0.009 \pm 0.005$. Overall, these results indicate the importance of excited state pumping to achieving pure single photon emission, even in situations in which there is only one quantum dot that can interact with the cavity

mode.

Finally, we note that in the photon antibunching experiments, the grating spectrometer was used as a monochromator to spectrally isolate the quantum dot emission, and had a throughput of $\approx$ 11 %. The output of the monochromator was coupled into single mode fiber and sent into the Hanbury-Brown and Twiss setup as described above, and the detected count rates on each of the two SPADs was $\approx 2\times10^4$ counts/s in the measurements from Fig. 4(e) in the main text. Overall, this detected count rate includes the collection efficiency of quantum dot emission into the NA = 0.4 lens ($\approx$ 48 %), the transmission of the photoluminescence setup ($\approx$ 42 %), the throughput of the monochromator ($\approx$ 11 %), coupling from the monochromator output into single mode fiber and throughput of the single-mode-fiber-based Hanbury-Brown and Twiss setup ($\approx$ 12 %), and the SPAD quantum efficiency ($\approx$ 20 %).

Supplementary Note 4: Comparison to single-photon sources created without optical positioning

For the purposes of comparison, in this section we present data from quantum dot single-photon sources in which quantum dot positioning was not employed (so that the position of the quantum dot with respect to bullseye cavity center was uncontrolled). The investigation of these devices was described in detail in Ref. 2, where spectroscopy, lifetime, and photon correlation measurements were presented. In Supplementary Fig. 3(a), we show an EMCCD image of a subset of the array of cavities investigated in Ref. 2, where the array has been illuminated by the 630 nm red LED. This EMCCD image reveals two new pieces of information. First, only one of twelve displayed devices shows an emission lobe near the center of the cavity, for which the collection efficiency is expected to be maximized. For this unpositioned sample, the maximum collection efficiency measured was ≈ 10 %, and the fraction of devices producing this efficiency was a couple of percent. Next, the quantum dot density in this sample is significantly higher than that studied in the current manuscript. While the density is still low enough so that only a single quantum dot can spatially and spectrally interact with a mode of the cavity, it is about two orders of magnitude larger than what we use in the positioned quantum dot devices. The background emission caused by these quantum dots, and in particular, their potential for supporting quasi-continuum states with broad emission bandwidths[3], may limit the purity of single-photon emission. Given that the yield for this sample is only a couple of percent, reducing the quantum dot density without locating the quantum dots prior to fabrication is impractical.

Spectroscopy and photon counting measurements from Ref. 2 further address these points. A typical photoluminescence spectrum under non-resonant pulsed excitation is shown in Supplementary Fig. 3(b). In contrast to the clean spectrum shown in the main text in Fig. 4(d), the spectrum of Supplementary Fig. 3(b) shows significant background emission attributed to feeding of the cavity mode by multi-excitonic states of nearby quantum dots. This emission can be expected to limit the purity of the single-photon source produced by spectrally isolating a single excitonic state, and indeed, the $g^{(2)}(\tau)$ measurement in Supplementary Fig. 3(c) shows a significant departure from $g^{(2)}(0) = 0$. While this measurement is of a device with a particularly high $g^{(2)}(0)$ value, in general, unpositioned devices studied in Ref. 2 showed $g^{(2)}(0) \gtrsim 15$ %. That being said, the discussion from the previous section indicates that even the drastically reduced quantum dot density used in the current work most likely needs to be supplemented by excited state pumping of the quantum dot in order to achieve $g^{(2)}(0) \approx 0$.

Supplementary Note 5: Electromagnetic simulations

As discussed in the main text and in Ref. 2, the bullseye cavity supports dipole-like resonant modes (shown in Supplementary Figs. 4(b) and (c)) that are well-suited for the creation of bright single-photon sources - a combination of relatively high Purcell-type radiative enhancement, efficient vertical light extraction from the semiconductor, and near-Gassian far-field for efficient collection into an optical fiber. These modes are strongly localized at the center of the cavity (the central intensity peak has a full-width at half-maximum of $\approx$ 100 nm), and a sequence of satellite peaks along the radial direction. Because the electric dipole coupling to a cavity mode is proportional to the squared electric field magnitude *at the dipole location*[6], we expect that the Purcell enhancement factor F_p, coupling efficiency η, and emitted polarization state will vary significantly with dipole position. An understanding of these parameters is not just important from a device performance perspective, but also provides information about the actual quantum dot location. We employ full-wave numerical electromagnetic simulations to investigate the sensitivity of the emission properties of our single-photon source to the location of the quantum dot within the bullseye cavity.

A. Purcell Factor and Collection Efficiency

Following Ref. 6, we use finite-difference time domain (FDTD) simulations to model the system as an electric dipole radiating inside a suspended bullseye cavity. The dipole is allowed to radiate with a short Gaussian pulse time dependence, and the electromagnetic field is allowed to evolve over a long time span. The steady-state electromagnetic field is recorded at all edges of the computational window, so that the total dipole radiated power P_{rad} can be determined. The Purcell factor can then be obtained as $F_p = P_{rad}/P_{hom}$, where P_{hom} is the dipole radiated power in a homogeneous medium[6]. We also record the power P_z emitted upwards in the $+z$ direction, which in a real setting is partially collected with a microscope objective with numerical aperture NA. The steady-state field recorded at a parallel plane above the bullseye cavity is used to calculate the emitted far-field, which is then integrated within an angular cone corresponding to a numerical aperture NA to yield the collection efficiency η_{NA}. Perfectly matched layers are used to simulate free-space above and below the cavity, so that effects related to the substrate are not taken into account. The dipole is assumed to be on the $z = 0$ plane (which corresponds to the center of the semiconductor membrane) as defined by the quantum dot growth process, and to have no z-components. The latter assumption is appropriate for epitaxially grown InAs dots, given their few nanometer vertical size, negligible compared to the membrane thickness[7].

In the circular geometry of the cavity, an electric dipole with moment $\mathbf{d}$ with arbitrary orientation placed anywhere in the cavity is equivalent to a dipole located on the x-axis with components along the x and y directions, corresponding to the radial and azimuthal components. This is illustrated in Supplementary Fig. 4(a). The symmetry of the problem allows a description of the electromagnetic fields supported by the cavity in terms of orthogonal, symmetric and anti-symmetric cavity eigenmodes with respect to the $y = 0$ plane ('h'-modes) and degenerate modes of the 90-degree rotated geometry ('v'-modes), as illustrated in Supplementary Figs. 4(b) and (c). An x-dipole will however only excite symmetric h-modes and anti-symmetric v-modes, and vice-versa is valid for a y-dipole; in other words, $\mathbf{d}\cdot\mathbf{E^h} = d_x \cdot E_x^h$ and $\mathbf{d}\cdot\mathbf{E^v} = d_y \cdot E_y^v$.

From ref. 6, the total power emitted by the dipole in the cavity is $P_{rad} \propto \sum_n |\mathbf{d}\cdot\mathbf{E^n}|^2$, where $\mathbf{d}$ is the dipole moment and $\mathbf{E^n}$ is the (normalized) electric field for mode n, evaluated at the dipole location. With the symmetry considerations above, we can write

$$P_{rad} \propto \sum_n \left|d_x \cdot E_x^{h,n}\right|^2 + \left|d_y \cdot E_y^{v,n}\right|^2 = |\mathbf{d}|^2 \left\{ \sum_n \left|\cdot E_x^{h,n}\right|^2 \cos^2\phi + \left|\cdot E_y^{v,n}\right|^2 \sin^2\phi \right\}, \tag{1}$$

where ϕ is the dipole orientation - the angle the dipole makes with respect to the x-axis - which we assume to be unknown. Equation (1) thus allows us to determine the dipole emitted power P_{rad} for a dipole positioned anywhere on the x axis, with arbitrary angle given by ϕ, just based on the E^h and E^v modes which are respectively excited by the x and y dipole components.

As such, we proceed to calculate P_{rad} and the collection efficiency $\eta_{0.4}$ for $NA = 0.4$ separately for x- and y-oriented dipoles located on the x-axis at varying distance x_0 from the cavity center. We then use eq.(1) to determine the range within which the quantities of interest can vary due to the (unknown) azimuthal dipole orientation ϕ. To verify that this procedure is valid, we also simulate the case $\phi = 45°$, and compare the collection efficiency with that obtained through eq. (1) and the $x-$ and $y-$ dipole solutions. As shown in Supplementary Fig. 5, the difference between the two types of calculations is $\lesssim 1$ % almost everywhere.

In Supplementary Figs. 6(a) and (b), we show the Purcell Factor F_p as a function of wavelength for x- and y-dipoles, respectively, located at varying positions along x. At a wavelength of 948 nm, the x dipole couples to the 'h' resonance shown in Supplementary Fig. 4(b), while the y-dipole couples to the degenerate 'v' mode in Supplementary Fig. 4(c), and the Purcell Factor F_p peaks for dipoles at the cavity center. For y-dipoles displaced from the center, F_p shows a sequence of satellite peaks observed for increasing distances, which contrasts with the x-dipole case. This can be understood based on the variation of of the v and h field profiles along the x-axis (Supplementary Figs. 4(b)-(c)), as $F_p \propto |E|^2$. A second resonance centered at 957 nm exists that is also excited by dipoles in both orientations, however displays considerably lower Purcell enhancement and collection efficiency (shown later). Supplementary Figs. 6(c) and (d) show the overall maximum and minimum achievable F_p, and

the shaded areas in Supplementary Fig. 6(e) correspond to overall allowed values of F_p as a function of dipole displacement, for three wavelengths around the resonance center. Essentially, these ranges correspond to the uncertainty in our knowledge of F_p due to lack of knowledge of the in-plane dipole orientation. Dotted white lines, on the other hand, correspond to the case $\phi = 45°$, which corresponds to an in-plane isotropic dipole.

Supplementary Figs. 7(a) and (b) show the overall maximum and minimum achievable collection efficiency $\eta_{0.4}$, and the shaded areas in Supplementary Fig. 6(c) correspond to overall allowed values of $\eta_{0.4}$ as a function of dipole displacement, for three wavelengths around the resonance center. White dotted lines are for the $\phi = 45°$ case. It is evident that the collection efficiency is a much slower function of both wavelength and dipole displacement than the Purcell factor. As a result, for the QD-cavity wavelength detuning of the device we focus on in the main text (1.6 nm), there is a $\approx \pm$ 250 nm range of dipole positions consistent with the experimentally observed collection efficiency (48 % $\pm$ 5 %) and Purcell Factor (≈ 3). The lack of knowledge about the QD dipole orientation ϕ prevents us from more precisely estimating the location of the emitter within the cavity. For example, if $\phi = 45°$, from Supplementary Figs. 6(e) and Supplementary Fig. 7(c) we can estimate that the dipole is located within 50 nm of the cavity center, in order to display the experimentally observed F_p and $\eta_{0.4}$.

We note however that the collection efficiency maximum is shifted with respect to the resonance center by approximately -5 nm, as can be seen in Supplementary Figs. 6(a)-(d) and Supplementary Figs. 7(a)-(b). This is due to far-field collection efficiency, which is actually asymmetric with respect to the resonance center, being higher by approximately 0.5 % at a maximizing blue-shifted wavelength. While this information is still not sufficient to pinpoint the quantum dot location based on our experimental data, it further corroborates our explanation that the relatively low observed Purcell factors can still exist with high collection efficiencies.

B. Polarization of the light emitted by a dipole embedded within a bullseye cavity

We now study the polarization properties of the light emitted by a dipole in the bullseye cavity. In particular, our goal is to understand the degree to which polarization-resolved measurements of the far-field intensity can be used to identify the dipole orientation, which in turn would enable more precise determination of the dipole location from Purcell enhancement and collection efficiency measurements.

The 'h' and 'v' bullseye cavity modes overall display major electric field components oriented in the x and y directions, respectively. This can be verified in two ways: plots of $|E_x|^2$ and $|E_y|^2$ for the 'v' mode in Supplementary Figure 8(a) show the former to be overall at least an order of magnitude larger than the latter; and the ratio $R_{xy} = \int_{S_{NA}} \mathbf{dS}|E_x|^2 / \int_{S_{NA}} \mathbf{dS}|E_y|^2$, where S_{NA} is the spherical surface corresponding to a $NA = 0.4$ cone, is calculated to be $R_{xy} = 3.47$. As such, we expect the far-field produced by a dipole at an arbitrary orientation characterized by the azimuthal angle ϕ to display some degree of polarization. This degree of polarization can in principle be resolved by introducing of a linear polarizer above the cavity and determining the variation of the transmitted power (collected into a 0.4 NA optic) with respect to the polarizer orientation.

To perform this calculation, we first note that the radial component of the far electric field is much smaller than the azimuthal and polar ones ($|\mathbf{E}_\rho| \ll |\mathbf{E}_\phi|, |\mathbf{E}_\theta|$ in spherical coordinates). We then assume that the collection cone is narrow enough that the field at the entrance of the collecting lens can be well represented as $\mathbf{E} = E_x\hat{\mathbf{x}} + E_y\hat{\mathbf{y}}$, where E_x and E_y are the x- and y-components of the far-field (in other words, we take E_x and E_y to be the transverse components of the far-field). This allows us to use Jones matrix formalism to estimate the power transmitted through the polarizer. We represent a polarizer oriented at an angle θ_p with respect to the x-axis with the Jones matrix

$$\mathbf{M} = \begin{bmatrix} \cos\theta_p & \sin\theta_p \\ -\sin\theta_p & \cos\theta_p \end{bmatrix} \begin{bmatrix} 1 & 0 \\ 0 & 0 \end{bmatrix} \begin{bmatrix} \cos\theta_p & -\sin\theta_p \\ \sin\theta_p & \cos\theta_p \end{bmatrix} = \begin{bmatrix} \cos^2\theta_p & -\sin\theta_p\cos\theta_p \\ -\cos\theta_p\sin\theta_p & \sin^2\theta_p \end{bmatrix} \tag{2}$$

The transmitted electric field $\mathbf{E_{out}} = \mathbf{ME}$ is, then,

$$\begin{bmatrix} E_x \\ E_y \end{bmatrix}_{out} = \begin{bmatrix} E_x\cos^2\theta_p - E_y\sin\theta_p\cos\theta_p \\ E_y\sin^2\theta_p - E_x\sin\theta_p\cos\theta_p \end{bmatrix}. \tag{3}$$

The transmitted power is proportional to $|\mathbf{E}|^2 = |E_x|^2 + |E_y|^2$. If the emitting dipole is at an arbitrary orientation, both 'h' and 'v' modes are produced in the cavity, so that, in the far-field, $\mathbf{E} = \alpha_h\mathbf{E^h} + \alpha_v\mathbf{E^v}$ ($\alpha_{h,v}$ represent the dipole coupling strength to the h and v modes). In this case, the resulting expression for the transmitted power consists of a sum of terms $E_k^iE_l^{j*}$, where $i,j \in \{h,v\}$ and $k,l \in \{x,y\}$. In determining the transmitted power, all of these terms are integrated over a portion of a spherical surface which represents the acceptance cone of the collection lens. Because of the cylindrical symmetry of the cavity, the x and y components of the 'h' and 'v' fields obey the following symmetry relations (as seen in Supplementary Figs. 8(b),(c),(d) and (f)): E_x^h and E_y^v are even in x and y; E_x^v and E_y^h are odd in x and y. Because the integration is performed symmetrically in the xy plane, any cross-term $E_k^iE_l^{j*}$ that results odd in x and y has no contribution to the power; these are cross terms with $\{i \neq j, k = l\}$

and $\{i = j, k \neq l\}$. We can thus write the integrands

$$|E_x|^2 = \cos^4\theta_p \left(\left|E_x^h\right|^2 + |E_x^v|^2\right) + \cos^2\theta_p \sin^2\theta_p \left(\left|E_y^h\right|^2 + |E_y^v|^2\right) - 2\cos\theta_p \sin^3\theta_p \Re\left\{E_x^h E_y^{v*} + E_x^v E_y^{h*}\right\} \tag{4}$$

and

$$|E_y|^2 = \sin^4\theta_p \left(\left|E_y^h\right|^2 + |E_y^v|^2\right) + \cos^2\theta_p \sin^2\theta_p \left(\left|E_x^h\right|^2 + |E_x^v|^2\right) - 2\cos^3\theta_p \sin\theta_p \Re\left\{E_x^{h*} E_y^{v} + E_x^{v*} E_y^{h}\right\}, \tag{5}$$

where the substitutions $\mathbf{E^{h,v}} \leftarrow \alpha_{\mathbf{h,v}} \mathbf{E^{h,v}}$ were done for simplicity. We use equations (4) and (5) to estimate the collected power that is transmitted through the polarizer, at any polarizer orientation angle . The visibility V can be determined from the maximum and minimum intensities with respect to the polarizer angle as

$$V = \frac{I_{max} - I_{min}}{I_{max} + I_{min}}, \tag{6}$$

where $I = \int_{S_{NA=0.4}} \mathbf{dS}\,|\mathbf{E}|^2$, where $S_{NA=0.4}$ is the spherical surface corresponding to the $NA = 0.4$ collection cone. Once again, the power radiated into $\mathbf{E^h}$ and $\mathbf{E^v}$ by a dipole located at an arbitrary position in the cavity depends on the dipole's orientation; and because the dipole orientation is not known, we can only determine the possible ranges of V at each dipole location. As such, we can only determine the range of achievable visibilities V at each dipole location. This is shown as a function of wavelength in Supplementary Fig. 9(s).

These plots indicate the non-monotonic dependence of the visibility on dipole location and orientation. As a result, a measurement of the visibility, taken together with measurements of the Purcell enhancement and collection efficiency, usually does not provide an unambiguous estimate of the dipole location.

For example, we have measured $V = 0.8$ for the device described in detail in the main text. Based on the quantum dot cavity detuning at the time of this measurement (-1.6 nm; lower panel in Supplementary Fig. 9(c)), we find that this visibility is consistent with multiple different locations for the dipole, including ≈ 100 nm away from the cavity center. While this is consistent with our estimate of dipole location based on the Purcell enhancement and collection efficiency measurements (< 250 nm from the bullseye center), it does not provide a significantly improved estimate of the dipole location. Additional measurement techniques (for example, spatially-resolved polarization-dependent far-field measurements) may be required to achieve a better estimate.

Supplementary References

---

[1] K. A. Serrels, E. Ramsay, P. A. Dalgarno, B. Gerardot, J. O'Connor, R. H. Hadfield, R. Warburton, and D. Reid, "Solid immersion lens applications for nanophotonic devices," Journal of Nanophotonics **2**, 021 854 (2008).
[2] S. Ates, L. Sapienza, M. Davanco, A. Badolato, and K. Srinivasan, "Bright single-photon emission from a quantum dot in a circular Bragg grating microcavity," IEEE Journal of Selected Topics in Quantum Electronics **18**, 1711–1721 (2012).
[3] M. Winger, T. Volz, G. Tarel, S. Portolan, A. Badolato, K. Hennessy, E. L. Hu, A. Beveratos, J. Finley, V. Savona, and A. Imamoglu, "Explanation of photon correlations in the far-off-resonance optical emission from a quantum - dot cavity system," Phys. Rev. Lett. **103**, 207 403 (2009).
[4] N. Chauvin, C. Zinoni, M. Francardi, A. Gerardino, L. Balet, B. Alloing, L. H. Li, and A. Fiore, "Controlling the charge environment of single quantum dots in a photonic-crystal cavity," Phys. Rev. B **80**, 241 306 (2009).
[5] A. Laucht, M. Kaniber, A. Mohtashami, N. Hauke, M. Michler, and J. Finley, "Temporal monitoring of nonresonant feeding of semiconductor nanocavity modes by quantum dot multiexciton transitions," Phys. Rev. B **81**, 241 302 (2010).
[6] Y. Xu, J. S. Vučković, R. K. Lee, O. J. Painter, A. Scherer, and A. Yariv, "Finite-difference time-domain calculation of spontaneous emission lifetime in a microcavity," J. Opt. Soc. Am. B **16**, 465–474 (1999).
[7] P. Michler, *Single Semiconductor Quantum Dots* (Springer Verlag, Berlin, 2009).
